# Detection of Zika Virus in Desiccated Mosquitoes by Real-Time Reverse Transcription PCR and Plaque Assay

**DOI:** 10.3201/eid2304.161772

**Published:** 2017-04

**Authors:** Kristen L. Burkhalter, Harry M. Savage

**Affiliations:** Centers for Disease Control and Prevention, Fort Collins, Colorado, USA

**Keywords:** Zika virus, viruses, arboviruses, real-time reverse transcription PCR, Vero cells, plaque assay, cold chain, virus detection, Culicidae, virus RNA, mosquitoes, virus persistence in mosquitoes

## Abstract

We assayed Zika virus–infected mosquitoes stored at room temperature for <30 days for live virus by using plaque assay and virus RNA by using real-time reverse transcription PCR. Viable virus was detected in samples stored <10 days, and virus RNA was detected in samples held for 30 days.

Laboratory detection of Zika virus in field-collected mosquitoes is often accomplished by using molecular methods, such as reverse transcription PCR (RT-PCR) or virus isolation protocols. Sample handling conditions recommended for these assays include maintaining a cold chain from field to laboratory to preserve live virus and mitigate degradation of virus RNA.

Ideally, mosquitoes should be stored and shipped to a diagnostic laboratory at a temperature of −70°C or below. However, in some locations, an optimal cold chain is difficult to maintain when shipping the specimens because of unavailability of a liquid nitrogen or dry ice supplier. We tested Zika virus–infected mosquitoes that had been stored at room temperature for <30 days by using real-time RT-PCR to determine the rate of recovery of virus RNA and Vero cell plaque assays to determine the rate of recovery of infectious virus.

## The Study

Female, laboratory-reared *Aedes aegypti* (REXD strain) mosquitoes were intrathoracically inoculated with 0.3 μL of ≈4.7 log_10_ PFU/mL of Zika virus (MR766 strain) as described (*1*). Inoculated mosquitoes were incubated for 7 days at 26°C and a relative humidity of 80% during a 16:8 h photoperiod, then killed by freezing overnight at −80°C. Nine mosquitoes were retained at −80°C until the end of the experiment to serve as controls (referred to as day 0). The remaining mosquitoes were kept in an open petri dish at room temperature (21°C) and a relative humidity of 50% during a 16:8 h photoperiod until collected. Six mosquitoes were collected every other day, from day 2 through day 30, and stored at −80°C until tested.

Mosquitoes were homogenized individually in 500 μL of cell culture medium/bovine albumin by vortexing with 1 copper-clad steel bead, and centrifuged for 3 min at 1,700 × *g*. We extracted virus RNA from a 100-μL aliquot of the resulting supernatant by using the QIAmp Virus BioRobot 9604 Kit and a BioRobot Universal System (QIAGEN, Valencia, CA, USA) according to the manufacturer’s protocol. Real-time RT-PCR (sensitivity limit 25 RNA copies) (*2*) was performed by using a Quantitect Probe RT-PCR Kit (QIAGEN) and primers specific for the envelope region of the Zika virus genome, according to the manufacturer’s instructions. Samples with a cycle threshold (C_t_) <38 were considered positive. In addition, day 0 control mosquitoes that had been stored at −80°C and all mosquitoes collected on days 2–30 were assayed for viable virus by using Vero plaque assay cell culture as described (*3*).

Vero cell plaque assay results for day 0 samples, which were frozen until testing and served as controls, had expected virus titers (range 6.3–6.8 log_10_ PFU/mL) (Table). Viable virus was recovered from 6/6 (100.0%) pools collected on day 2, 2/6 (33.3%) pools collected on day 4, 1/6 (16.6%) pools collected on day 6, and 1/6 (16.6%) pools collected on day 10. Titers of viable virus recovered from individual desiccated mosquitoes at days 2–10 ranged from 1.69 to 2.6 log_10_ PFU/mL (Table). The remaining replicates from days 4, 6, and 10, and all replicates from day 8 and days 12–30 did not produce plaques. A similar study with dried Zika virus stock stored at room temperature for 4.5 days showed that virus was still infectious after reconstitution at 3.5 days (*4*), which correlates with our findings for desiccated mosquitoes.

**Table:** Zika virus titers for infected mosquitoes stored at room temperature (21°C) and collected every other day for 2–30 days and control samples*

Day of collection	No. samples with live virus/no. tested (%)	Mean titer, log_10_ PFU/mL (95% CI)
0	9/9 (100.0)	6.62 (6.52–6.72)
2	6/6 (100.0)	2.3 (2.06–2.54)
4	2/6 (33.3)	1.85 (0.63–3.07)
6	1/6 (16.6)	1.69
8	0/6 (0)	NA
10	1/6 (16.6)	2.5
12–30	0/6 per day (0)	NA

*Control samples (day 0) were stored at −80°C until testing. NA, not applicable.

Although virus titers of desiccated mosquitoes had been reduced, evidence of residual infectivity indicates that precautions, such as use of proper personal protective equipment and appropriate biosafety protocols, are required when handling field-collected samples regardless of storage conditions because these samples might contain viable virus. The large reduction of infectivity in desiccated samples collected on days 2–10 and the complete reduction of infectivity in samples beyond day 10 confirm that a cold chain is required if infectious virus retrieval is the goal of the field collection effort.

Maintaining a cold chain is less critical for detecting Zika virus RNA in mosquito pools. Real-time RT-PCR C_t_ values for day 0 control samples ranged from 23.9 to 29.1 (Figure). Although C_t_ values of desiccated samples increased over the 30-day holding period, all samples from each time point were positive (C_t_ <38), with the exception of 1 sample from day 18 from which virus RNA could not be detected (Figure). Stability of virus RNA from either mosquito samples or lyophilized virus held at room temperature has been demonstrated for other arboviruses, including West Nile, chikungunya, yellow fever, and Venezuelan equine encephalitis viruses (*5**–**7*). Future evaluations that include variable temperature, lighting conditions, and humidity would elucidate how diverse conditions encountered in the field affect recovery of Zika virus RNA.

**Figure:**
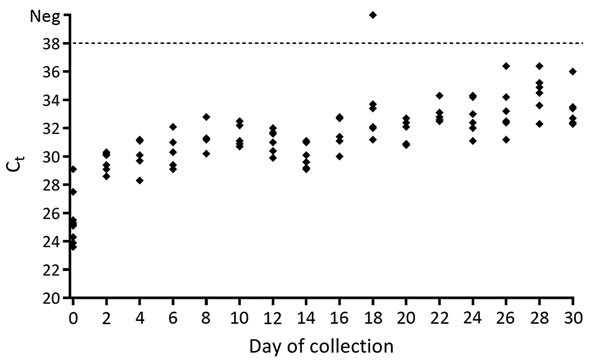
Real-time reverse transcription PCR cycle threshold (C_t_) values for mosquitoes infected with Zika virus and stored at room temperature (21°C) for 2–30 days and for control samples (day 0) stored at −80°C until testing. Samples with a C_t_ <38 (dashed line) were considered positive. Each diamond indicates a mosquito pool. Neg, negative.

## Conclusions

Results from this study suggest that, although Zika virus RNA in samples kept at room temperature degrades with time, qualitative detection by real-time RT-PCR is possible for up to 30 days. Although agencies should strive to implement recommended protocols and maintain a cold chain, this is not always possible when performing field-based entomologic arbovirus surveillance, particularly in remote areas where dry ice and ultralow freezers are absent. If the goal of a surveillance program is to determine the infection rate in mosquito populations as efficiently as possible, and is not focused on recovery of live virus, storage and shipment from the field to the testing laboratory under less than optimal conditions would be sufficient for virus detection by using real-time RT-PCR.
